# An industrial scale process for the enzymatic removal of steryl glucosides from biodiesel

**DOI:** 10.1186/s13068-015-0405-x

**Published:** 2015-12-21

**Authors:** Salvador Peiru, Andres Aguirre, Florencia Eberhardt, Mauricio Braia, Rodolfo Cabrera, Hugo G. Menzella

**Affiliations:** Genetic Engineering and Fermentation Technology, Facultad de Ciencias Bioquímicas y Farmacéuticas, Universidad Nacional de Rosario-Conicet, Suipacha 531, Rosario, 2000 Argentina; Keclon S.A., Tucuman 7180, Rosario, 2000 Argentina; Unitec Bio S.A., Batalla del Quebracho s/n, Pto. Gral, San Martín, 2202 Argentina

**Keywords:** Synthetic biology, Green chemistry, Biofuels

## Abstract

**Background:**

Biodiesels produced from transesterification of vegetable oils have a major quality problem due to the presence of precipitates, which need to be removed to avoid clogging of filters and engine failures. These precipitates have been reported to be mostly composed of steryl glucosides (SGs), but so far industrial cost-effective methods to remove these compounds are not available. Here we describe a novel method for the efficient removal of SGs from biodiesel, based on the hydrolytic activity of a thermostable β-glycosidase obtained from *Thermococcus litoralis*.

**Results:**

A steryl glucosidase (SGase) enzyme from *T. litoralis* was produced and purified from *Escherichia coli* cultures expressing a synthetic gene, and used to treat soybean-derived biodiesel. Several optimization steps allowed for the selection of optimal reaction conditions to finally provide a simple and efficient process for the removal of SGs from crude biodiesel. The resulting biodiesel displayed filterability properties similar to distilled biodiesel according to the total contamination (TC), the cold soak filtration test (CSFT), filter blocking tendency (FBT), and cold soak filter blocking tendency (CSFBT) tests. The process was successfully scaled up to a 20 ton reactor, confirming its adaptability to industrial settings.

**Conclusions:**

The results presented in this work provide a novel path for the removal of steryl glucosides from biodiesel using a cost-effective, environmentally friendly and scalable enzymatic process, contributing to the adoption of this renewable fuel.

**Electronic supplementary material:**

The online version of this article (doi:10.1186/s13068-015-0405-x) contains supplementary material, which is available to authorized users.

## Background

Biodiesels comprise a mixture of fatty acid methyl esters synthesized via transesterification of oils and fats with short chain alcohols. Being mostly produced from vegetable oils, including those from soybean, palm, sunflower, rapeseed, jatropha, and others, they are promising renewable alternatives for petroleum-based fuels. Biodiesels can be used alone or in blends in traditional fuel-burning engines, have a higher flash point and reduced sulfur emissions, and are less toxic than petroleum-based fuels. Currently, obligatory and voluntary biodiesel mandates are set in more than 60 countries, many which together constitute high-consuming regions like the European Union and the United States [1]. Driven by these policies and goals for renewable energy around the world, global biodiesel production is projected to continue its rapid increase and reach more than 40 billion liters by 2020 [2].

Unfortunately, transesterification of oil produces unwanted side products that form sediments that may cause engines to fail, which affects the acceptance of biodiesel as an alternative fuel. Steryl glucosides (SGs), present in different biodiesels at concentrations ranging from 10 to 300 ppm [3, 4], have been identified as the major component of such sediments [5–9]. Naturally occurring acylated SGs are soluble in oil, but during the esterification process they are converted to nonacylated SGs, which are insoluble in biodiesel at low temperatures [7, 10, 11]. Particles composed of clumped SG molecules also promote aggregation or precipitation of other compounds in the biodiesel, such as saturated monoacyl glycerides (SMGs), which further reduces the fluidity of biodiesel and increases the likelihood of clogging [12, 13]. Additionally, the presence of this sediment causes several complications during biodiesel production, which increases costs [7, 12, 14, 15].

Biodiesel containing SGs appears hazy, and a white sediment typically forms during its storage. These conditions often prevent the product from meeting quality standards for contamination and filterability. Thus, the selective removal of SGs may produce biodiesels of superior quality, helping these renewable fuels to be adopted by consumers and therefore contributing to the development of the biodiesel industry. Currently, the only available method capable of completely removing SGs from biodiesel is distillation, an energy-intensive and expensive process [16, 17]. This circumstance therefore reduces the cost efficiency and net energy gain of biodiesel production [4, 18, 19]. Other methods, including cold soak filtration, centrifugation and filtering through diatomaceous earth, magnesium silicate and bleaching earth have shown to have limited utility in providing biodiesels with high quality [4, 12, 18, 20].

**Fig. 1:**
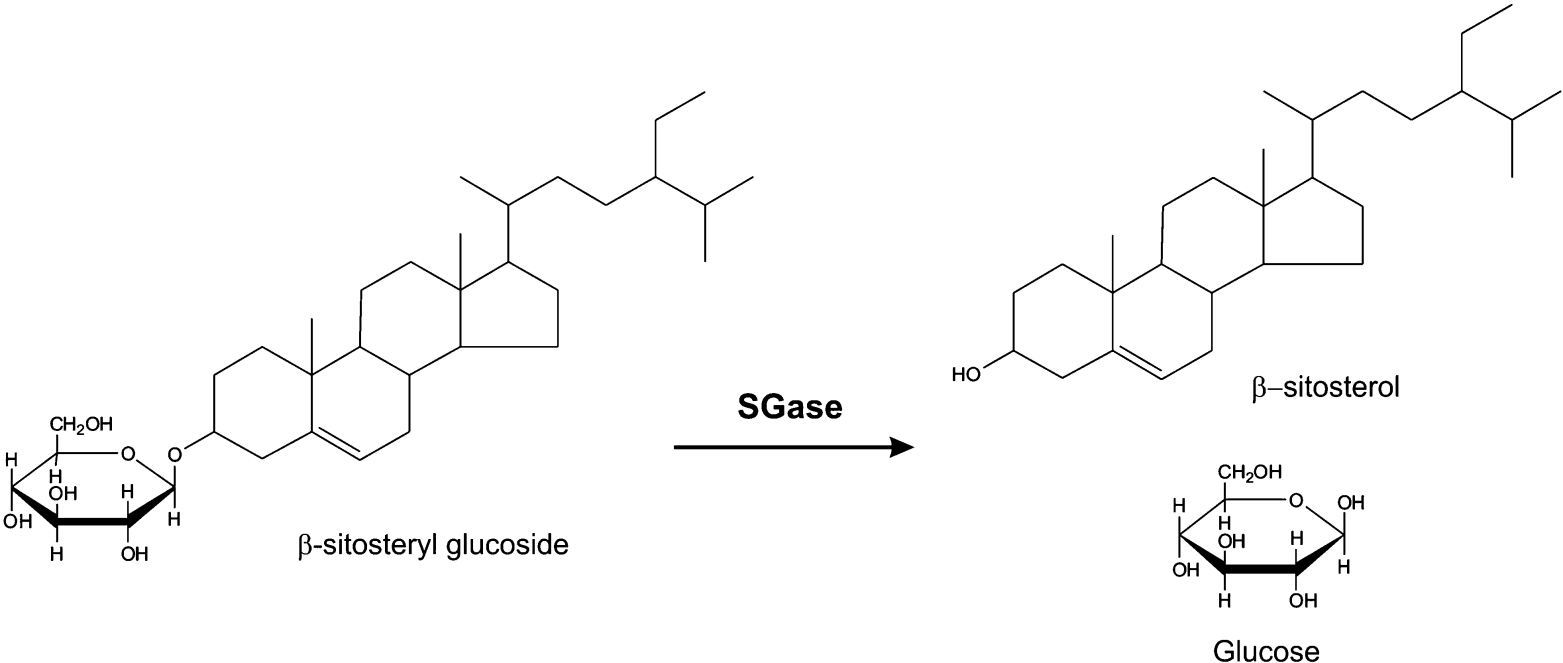
Schematic representation of the SGase reaction, showing the hydrolysis of β-sitosteryl glucoside, the most abundant SG in soybean oil

We hypothesized that an enzyme with steryl glycosidase activity (SGase) could be used to hydrolyze SGs, producing glucose and a sterol (Fig. 1). The generated sterols would be completely soluble in biodiesel, while the glucose would be eliminated subsequently during the water-washing steps after transesterification. Here we describe the identification, characterization, and heterologous production of an enzyme capable of reducing SGs levels from biodiesel below detection limits, and we present a scalable method for using the enzyme in biodiesel production plants.

## Results

### Gene selection, design, and expression vector construction

In previous work, we identified and characterized a family of thermostable enzymes capable of hydrolyzing SGs in both aqueous media and water/biodiesel emulsions [19]. The use of thermostable enzymes is desirable for an enzymatic treatment of biodiesel, since SGs are mostly insoluble at room temperature [21]. LacS, a β-glucosidase from *Sulfolobus solfataricus*, was the most efficient enzyme of this family when enzymatic reactions were performed in the presence of 0.9 % polyglycerol polyricinoleate (PGPR), a powerful water-in-oil emulsifier. The significant improvement in the activity of LacS in the presence of emulsifiers may reflect constraints of the enzyme reaching the SGs located at the water/biodiesel interface, possibly due to the location of the enzyme inside the water phase. Unfortunately, the use of these amounts of emulsifier in such an industrial biodiesel-cleaning process would exceedingly increase its cost. Thus, we started a new screen for β-glucosidases with enhanced performance in water/biodiesel biphasic systems and with a capacity to work in the absence of emulsifying agents. We initially focused our search on thermostable enzymes with features that promised better performance in water/biodiesel emulsions: tolerance to organic solvents, specificity to hydrophobic aglycones, and/or natural associations with membranes.

The initial screening for thermophilic β-glucosidases with specificity to hydrophobic substrates led us to an enzyme from *Pyrococcus horikoshii* (BGPh) that shares a 46 % similarity to LacS and that exhibits a remarkable tendency to hydrolyze β-glucosides with long alkyl chains [22]. Moreover, experimental data suggest that this enzyme is associated with the bacterial membrane, both when its activity is analyzed in membrane fractions of *Pyrococcus*, and when it is heterologously expressed in *Escherichia coli*. Therefore, the enzyme appears to have affinity for hydrophobic surfaces. Structural analysis of BGPh shows a hydrophobic mound at the end of the active site, which is surrounded by a thick ring of positively charged amino acids [23]. These features of the molecular surface suggest that the hydrophobic mound can enter the membrane layer, agreeing with the experimental evidence of its membrane localization. The surface structure of the membrane-bound region of BGPh differs markedly from that of the corresponding region of LacS, which is water soluble.

Thus, we hypothesized that BGPh could function as an SGase in the absence of emulsifiers. Expecting to widen the range of candidates, we selected six additional sequences that were most similar to BGPh, as identified from BLAST analysis (Table 1). The seven protein sequences were codon optimized for expression in *E. coli* using the OptimumGene™ algorithm (GenScript). The resulting DNA sequences were synthesized with an NdeI restriction site overlapping the ATG start codon and an EcoRI site situated downstream of the stop codon. The DNA was cloned into the same sites of the T7-based pET28a expression plasmid (Novagen) to obtain an N-terminal fusion to a His_6_ tag.

**Table 1:** Enzymes tested in this study and their homology to BGPh

Protein (GenBank accession no.)	% Homology to BGPh	Synthetic gene GenBank accession no.
BGPh *Pyrococcus horikoshii* (WP_010884453.1)	100	KP772234
BGPf *Pyrococcus furiosus* (WP_011011560.1)	96	KP772235
BGTl *Thermococcus litoralis* (WP_004069094.1)	92	KP772236
BGTk *Thermococcus kodakarensis* (WP_011250778.1)	91	KP772237
BGTs *Thermococcus sibiricus* (WP_015848865.1)	90	KP772238
BGAb *Aciduliprofundum boonei* (WP_008082059.1)	84	KP772239
BGTb *Thermococcus barophilus* (WP_013467240.1)	81	KP772240

### Gene expression, enzyme purification, and activity assays

Recombinant plasmids were transformed into the *E. coli* BL21(DE3) strain to assess expression. Cell cultures in LB medium were induced by the addition of IPTG when the OD_600_ reached 0.5. Cells were harvested after 5 h. Analysis of soluble and insoluble fractions of cell lysates by SDS-PAGE showed that only BGTl, BGTb, and BGTs were successfully expressed. Despite the use of Triton X-100 during cell lysis, only BGTl could be obtained in the soluble fraction in sufficient quantities for further analysis. Strategies to increase the fraction of soluble protein, such as changes in the gene expression system (promoters, vectors, codon optimization algorithms, host strains, culture conditions, and so forth) and the use of solubilization protocols, were unsuccessful (data not shown).

**Fig. 2:**
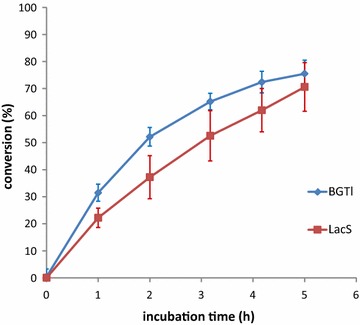
A time course of SGase activity (expressed as conversion %) of BGTl and LacS in aqueous buffer. Reactions were performed at 70 °C in phosphate buffer at pH 6.5 for BGTl and citrate buffer at pH 5.5 for LacS. 14 µg of enzyme/mL of buffer spiked with 100 ppm SGs was used. SGase hydrolysis was measured with a coupled enzymatic fluorescence assay. *Error bars* show the standard deviation of three independent assays

Next, BGTl was prepared from a 1 L culture and purified using a Ni^2+^-NTA affinity resin. The protein preparation was tested for SGase activity against a standard mixture of β-sitosteryl glucoside, campesteryl glucoside, stigmasteryl glucoside, and avenasteryl glucoside (56:25:18:1), a typical composition of SGs found in soybean crude biodiesel. The assay was performed in aqueous media as previously described, where the extent of SG hydrolysis is quantified by determining fluorometrically the formation of glucose through a coupled enzymatic assay [19]. Tests were run in parallel with LacS, and both showed a comparable ability to hydrolyze SGs under the assay conditions (Fig. 2).

### Evaluation of steryl glucosidase activity in biodiesel

**Fig. 3:**
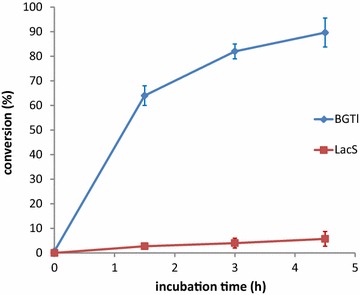
A time course of SGase activity (expressed as conversion %) of BGTl and LacS in distilled biodiesel. Reactions were performed in biodiesel/water mixtures (100:15) at 70 °C in 50 mM phosphate buffer (pH 6.5) for BGTl and 50 mM citrate buffer pH 5.5 for LacS. 14 µg of enzyme/g of biodiesel spiked with 100 ppm SGs was used. *Error bars* show the standard deviation of three independent assays

The main goal of this work was to obtain a new SGase capable of efficiently hydrolyzing SGs in water/biodiesel biphasic systems while avoiding the use of emulsifiers. Thus, we next tested BGTl in distilled soybean oil-derived biodiesel that was free of SGs and was supplemented with 100 ppm of SGs, an amount normally found in industrial crude biodiesel. For this assay, 0.18 mg of purified enzyme in 1.95 mL of the corresponding buffer was added to 13 g of biodiesel prepared as described above. LacS, an emulsifier-dependent SGase enzyme, was used as a control. The mixtures were incubated for up to 4.5 h at 70 °C with stirring, and the glucose generated from the hydrolysis of SGs was quantified by the coupled enzymatic assay. Figure 3 shows that about 6 % of the total content of SGs was hydrolyzed under the tested conditions using LacS, while most of the added SGs were hydrolyzed when using BGTl. This experiment was also performed using crude soybean biodiesel containing 75 ppm SGs, and similar results were obtained (data not shown).

The great improvement of SG removal from biodiesel achieved by BGTl compared to LacS could be due to its predicted affinity to hydrophobic surfaces. Molecular modeling of BGTl based on reported crystallographic data from BGPh confirms the presence of a hydrophobic patch similar to the one present in BGPh. A bias in the distribution of charged residues gives rise to a positive electrostatic potential surrounding the hydrophobic patch. These combined features drive the protein towards the water:biodiesel interface (Additional file 1) [23]. In previous work, we determined by measuring β-glucosidase activity that LacS is located in the water fraction of water/biodiesel mixtures [19]. To determine the location of BGTl in this system, we performed a similar experiment, using LacS as a control. For this assay, 0.18 mg of each purified enzyme in 1.95 mL of the corresponding buffer was added to 13 g of distilled biodiesel and stirred for 10 min. After mixing the phases, 1 vol of buffer was added and vortexed, and once the aqueous phase settled, a sample was taken to measure the recovered β-glucosidase activity. Table 2 shows that only 22 % of the activity could be recovered in BGTl samples, while under identical conditions the activity could be almost fully recovered from the aqueous phase of samples containing LacS. However, when the same experiment was performed in the presence of 1 % Triton X-100 in the added buffer, β-glucosidase activity recovered in BGTl samples increased up to 80 % of the initial activity (Table 2). This observation suggests that BGTl preferentially localizes to the water/biodiesel interface and would explain the different performances of LacS and BGTl during SG hydrolysis in biodiesel.

**Table 2:** BGTl and LacS localization in water/biodiesel biphasic systems

β-Glycosidase activity recovered (%)		
	Control	+Triton X-100
BGTl	22 ± 1	80 ± 4
LacS	99 ± 1	99 ± 1

Standard deviations were calculated on three independent assays

**Fig. 4:**
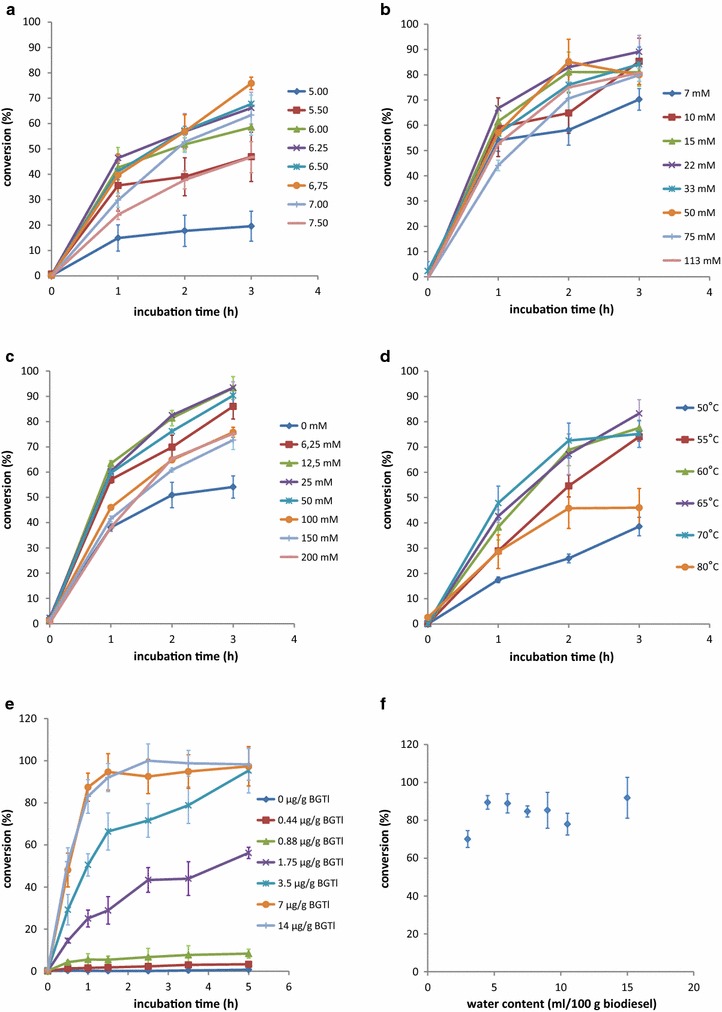
Optimization of the enzymatic process. Influence of reaction factors on the efficiency of BGTl-mediated hydrolysis of SGs (expressed as conversion %) in commercial soybean oil biodiesel/water mixtures. Experimental conditions: **a** 100:15 biodiesel/water mixtures, 5 µg of enzyme/g biodiesel, 70 °C, and 50 mM phosphate or citrate buffers of different pHs, **b** 100:15 biodiesel/water mixtures, 5 µg of enzyme/g biodiesel, 70 °C, phosphate buffer (pH 6.75) at different concentrations, **c** 100:15 biodiesel/water mixtures, 5 µg of enzyme/g biodiesel, 20 mM phosphate buffer (pH 6.75), 70 °C, and different concentrations of NaCl, **d** 100:15 biodiesel/water mixtures, 5 µg of enzyme/g biodiesel, 20 mM phosphate buffer (pH 6.75), 20 mM NaCl, at different temperatures, **e** 100:15 biodiesel/water mixtures, 20 mM phosphate buffer (pH 6.75), 20 mM NaCl, 65 °C, and different concentrations of BGTl, **f** biodiesel/water mixtures with different water content, 5 µg of enzyme/g biodiesel, 20 mM phosphate buffer (pH 6.75), 20 mM NaCl, 65 °C. *Error bars* show the standard deviation of three independent assays

To establish the optimal conditions for the enzymatic process on an industrial scale, we tested the influence of different factors, including pH, buffer composition, water/biodiesel ratio, reaction temperature, and enzyme concentration, on the efficiency of BGTl-mediated hydrolysis of SGs in biodiesel. Assays were performed using an industrial sample of crude soybean biodiesel containing 75 ppm SGs in 20 mL vials with magnetic stirring. Figure 4a illustrates the behavior of BGTl at different pHs, adjusted with citrate or phosphate buffers. The results show that the enzyme prefers pH values in the range of 5.5–7.5, and it exhibits a maximum performance around 6.75 using phosphate buffer. Different phosphate concentrations were analyzed to establish the minimum requirement for the process. Figure 4b shows that the phosphate concentration could be reduced to 10 mM without altering the enzyme performance. In the same way, the optimal concentration of NaCl was analyzed. Figure 4c shows that the best reaction conditions were observed at values between 6 and 50 mM, with an optimal concentration being around 20 mM NaCl. The effect of temperature on the hydrolysis of SGs by BGTl is illustrated in Fig. 4d. The data show that the enzyme displays a maximum activity at 65 °C. Finally, we assayed the minimal concentration of BGTl necessary to remove SGs completely from biodiesel. Figure 4e shows that 7 µg of enzyme per gram of biodiesel is enough to achieve this goal within 2 h, a reasonable time for an industrial process.

The previous experiments were tested with an arbitrary proportion of water, fixed at 15 mL per 100 g of biodiesel. Because less water in an industrial process minimizes waste, we tested the SGase reaction with varying quantities of buffer in crude biodiesel (Fig. 4f). We found that the water content required to obtain full hydrolysis of SGs could be reduced to 4.5 mL per 100 g of biodiesel while maintaining the optimal performance of the enzyme.

The optimal temperature and pH in a water/biodiesel mixture might differ from those observed in an aqueous reaction because of protein instability caused by an interaction with the organic solvent in the biphasic system. This explanation appears to be the case for the optimal temperature in a water/biodiesel mixture, which is far below the optimal value of 85 °C for reactions in buffer. The decrease in enzymatic activity of β-glucosidase recovered from the aqueous phase of the biphasic system at temperatures higher than 70 °C suggests protein denaturation (Additional file 2).

### Large-scale enzymatic treatment

**Fig. 5:**
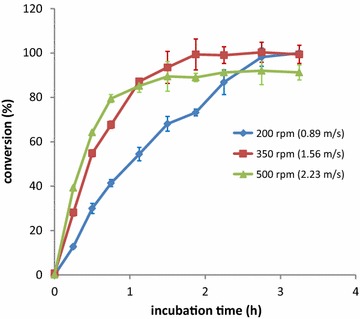
Effect of agitation on BGTl-mediated hydrolysis of SGs in a 15 L reactor. A time course of the hydrolysis of SGs (expressed as conversion %) in commercial biodiesel containing 65 ppm of SGs/water mixtures (100:4.5) using 7 mg of enzyme/kg biodiesel at 65 °C in 20 mM phosphate buffer pH 6.75 and 20 mM NaCl and at different agitation speeds. *Error bars* show the standard deviation of three independent assays

To validate the enzymatic process for biodiesel plants, we next conducted experiments in a 15 L reactor that had a 3:1 height/diameter ratio and was equipped with three six-blade Rushton type impellers and four baffles. The impeller diameter was 8.5 cm and the impeller spacing was 13 cm. This reactor was chosen because of its geometrical similarity to a large reactor available in a biodiesel plant, the similarity which facilitates scaling up strategies. The temperature was set at 65 °C and the reactor was loaded with 10 L of crude biodiesel containing 65 ppm of SGs, 0.45 L of 20 mM phosphate buffer at pH 6.7, 20 mM NaCl, and 7 mg of BGTl enzyme/kg of biodiesel. Figure 5 shows a time course of the reaction for three independent experiments that varied the speed of agitation. The optimal results were obtained when the agitation speed was set at 350 rpm (impeller tip speed 1.56 m/s), and the SGs were completely hydrolyzed within 2 h. Lower agitation speeds extended the time required to eliminate the SGs, while agitation speeds above 350 rpm yielded incomplete hydrolysis. As observed in the experiments to optimize temperature, analysis of protein stability revealed that the activity of BGTl decreases markedly at an agitation speed of 500 rpm (Additional file 3). This result indicates a limit for the rate of mixing due to protein instability at a fixed working temperature.

**Fig. 6:**
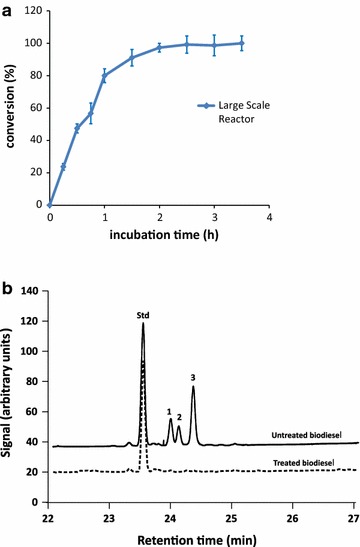
BGTl-mediated removal of SGs in a 20 ton industrial reactor. **a** A time course of the hydrolysis of SGs (expressed as conversion %) in a commercial biodiesel containing 75 ppm of SGs/water mixture (100:4.5) using 7 mg of enzyme/kg biodiesel at 65 °C in 20 mM phosphate buffer pH 6.75 and 20 mM NaCl and at 43 rpm. Error bars show the standard deviation of three independent assays. **b** SPE-GC-FID analysis of treated biodiesel samples. Traces are shifted on y axis for clarity. Peaks are labeled as follows: Std, cholesteryl glucoside standard; *1* campesteryl glucoside; *2* stigmasteryl glucoside and *3* β-sitosteryl glucoside

The process was finally scaled up to 20 ton based on a constant impeller tip speed approach, as this approach has been reported to provide the best scale-up criterion for liquid–liquid dispersions in stirred-tank reactors like the ones used here [24, 25]. The large-scale reactor had a height:diameter ratio of 3.5:1 and contained three six-blade Rushton type impellers (a diameter of 68 cm) and four baffles. The mixture of biodiesel, buffer, and enzyme was prepared as described above, the temperature was maintained at 65 °C, and the agitation speed was set at 43 rpm (impeller tip speed of 1.53 m/s). Initial SGs content on the biodiesel treated was 75 ppm. Figure 6a shows that the time course for the reaction at a large-scale mirrors the results obtained for the reaction at 15 l, indicating the success of the scaling up strategy.

A SPE-GC-FID analysis of SGs was performed on treated and untreated samples to support the fluorometrically obtained data. No signal was observed in the chromatograms for any of the three major SG present in the samples (Fig. 6b), confirming the effectiveness of the treatment in a multi-ton scale process.

### Impact on quality tests of biodiesel

Biodiesel must meet different quality standards as specified by different countries. Although none of these specifications stipulates a value limiting the content of SGs, biodiesel containing concentrations of SGs above 20 ppm usually fail to meet the standards of the most common tests of filterability [8, 12, 26]. However, the actual influence of SGs in plugging filters is still controversial, and the formation of precipitates is also attributed to the presence of SMGs [27]. SGase-treated biodiesel represents excellent material with which to specifically evaluate the influence of SGs in quality tests, since its concentration has been reduced below 3 ppm without other components present in the biodiesel matrix being altered. Therefore, we assessed dry SGase-treated soybean biodiesel in parallel with untreated samples (containing 75 ppm SGs) in the following tests: total contamination (TC), the cold soak filtration test (CSFT), filter blocking tendency (FBT), and cold soak filter blocking tendency (CSFBT). Our results are shown in Table 3, and they reflect a great improvement in biodiesel performance in all of the tests, possessing values far below the limits specified for each test. Moreover, SGase-treated biodiesel behaved similarly to distilled biodiesel even for the stringent CSFBT. It is worth mentioning that the content of SMGs in all soybean biodiesel samples was 0.1 %, indicating that, in the absence of SGs, even such high concentrations of SMGs do not affect the quality of biodiesel.

**Table 3:** Biodiesel quality test results

Sample	TC (ppm)	CSFT (s)	FBT	CSFBT
Untreated biodiesel	60 ± 4.5	458 ± 43	4.3 ± 1.0	6.5 ± 1.0
SGase-treated biodiesel	0.5 ± 0.2	80 ± 3	1.0 ± 0.0	1.0 ± 0.0
Distilled biodiesel	0 ± 0.0	70 ± 2	1.0 ± 0.0	1.0 ± 0.0
Specification (max)	24	360	1.8	1.8

Standard deviations were calculated on three independent samples

## Discussion

It has been reported that the presence of SGs originates problems of fluidity in both pure biodiesel and biodiesel blends [8, 12, 16, 26]. Particles containing SGs lead to the clogging of filters, causing engines that use this fuel to stop functioning. Thus, the removal of these compounds from biodiesel is necessary, but efficient and cost-effective methods have not been available [4, 12].

Recently, we identified LacS, an enzyme capable of hydrolyzing β-sitosteryl glucoside, campesteryl glucoside, and stigmasteryl glucoside, the most common SGs carried over from the vegetable oils used as a feedstock to produce biodiesel [19]. To significantly remove SGs from biodiesel/water mixtures, LacS requires the presence of emulsifiers. This requirement renders the process impractical at an industrial scale because of costs, possible contamination of the resulting biodiesel with traces of the emulsifier, and losses associated with the amphipathic nature of the additive. We hypothesized that enzymes that naturally work at water/lipid interfaces and that possess specificity for glucose conjugated to hydrophobic aglycones may have better access to amphipathic molecules such as SGs, helping us to circumvent the problems of LacS. An in silico search led us to a family of β-glycosidases gene sequences related to BGPh from *P. horikoshii*, a membrane-associated enzyme with the ability to hydrolyze glucosides with long alkyl chains [22]. A codon optimized version of a homologous gene from *T. litoralis*, called BGTl, proved to be the best candidate because of the high expression levels that were achieved in *E. coli*.

BGTI efficiently hydrolyzed SGs in the absence of emulsifiers. GC-FID analysis showed that the enzyme was equally efficient in hydrolyzing β-sitosteryl glucoside, campesteryl glucoside, and stigmasteryl glucoside, the three main types of SGs present in biodiesel derived from soybean oil. Optimization of working conditions led us to reduce the amount of water used in the hydrolysis to 4.5 % of the volume of treated biodiesel, which considerably lessened the amount of wastewater generated. Additionally, the concentration of ions present in the hydrolysis buffer was reduced to yield a biodiesel that met the requirements of ASTM standard D6751 and CEN standard EN14214.

The process was successfully scaled up to 20 ton using a stirred-tank reactor, in which 7 g of BGTI per ton of biodiesel completely eliminated the 75 ppm of SGs present in the sample within about 2 h. Currently, a continuous process to streamline the insertion of this enzymatic treatment in standard biodiesel plants is under development in our laboratory. A continuous stirred-tank reactor followed by a static separator are inserted in biodiesel production scheme after the aqueous washing step that follows the transesterification process, where the reaction conditions meet those required for the optimal activity of BGTI, including pH (5.5–7.5) and temperature (65 °C) [28]. After separation, biodiesel phase is dried as usual. As water phase and interphase are recycled in the continuous process, the enzymatic treatment implies no biodiesel losses. This contrasts with the >3 % inherent losses of the distillation process, or the 1.27 % losses recently reported for SGs removal using magnesium silicate [20]. Based on results obtained in large-scale fermentation experiments that will be published elsewhere, we anticipate that the manufacturing cost of the dose of enzyme require to treat one ton of biodiesel will be lower than $1. This cost is far below the current cost of distillation, estimated to be between $20 and $35 per ton in a survey made by us among the largest biodiesel producers in Argentina.

Although SGs are not measured directly in biodiesel on a routine basis due to technical difficulties, their presence has a deep impact on quality tests, including TC, CSFT, FBT, and CSFBT [8, 12, 16, 26]. The performance of the biodiesel treated with BGTI is in all of the cases comparable to that of distilled biodiesel, indicating the outstanding efficiency of our enzymatic process in providing a biodiesel of superior quality. Interestingly, the results of the tests were obtained with biodiesels containing 0.1 % of SMGs (0.65 % of total monoacyl glycerides), which have been considered major contaminants causing the formation of precipitates. Our results support recent observations suggesting that SGs are the main cause of precipitates [12, 16, 26], diminishing the relevance of SMGs as a biodiesel contaminant. As previously suggested, the presence of SMGs in biodiesel precipitates would thus be a consequence of the presence of SGs acting as nucleating agents to form such particles [13]. The enzymatic process presented here is the first report of an affordable biodiesel treatment capable of reducing SGs content in a standard commercial soybean biodiesel to levels required to pass stringent quality tests like FBT and CSFBT.

## Conclusion

We have developed an efficient process for the enzymatic removal of SGs from biodiesel, which represents an attractive alternative to improve the quality of this fuel. The process was validated on an industrial scale and the treated biodiesel largely complies with the most stringent filtration tests. This novel technology improves the quality of vegetable oil-derived biodiesel in a cost-effective manner and therefore should facilitate the global adoption of this renewable fuel.

## Methods

### General

Enzymes were obtained from New England Biolabs (USA) and used as recommended. DH5α and BL21(DE3) *Escherichia coli* strains were made chemically competent with a kit from Zymo Research (USA). DNA sequencing was performed on an ABI 3730 DNA analyzer (Applied Biosystems, USA) according to the manufacturer’s recommended protocol.

Commercial crude soybean biodiesel samples were obtained from Unitec Bio S.A. (Pto. Gral. San Martín, Argentina). Distilled soybean biodiesel was obtained from Intertek Argentina (Rosario, Argentina). A SGs standard was purchased from Matreya LLC (Pleasant Gap, PA, USA), containing a mixture of β-sitosteryl glucoside, campesteryl glucoside, stigmasteryl glucoside, and avenasteryl glucoside (56:25:18:1). The *p*-nitrophenyl *β*-d-glucopyranoside (pNPG), ATP and NADP^+^, the enzymes HK and G6PDH and other reagents were purchased from Sigma-Aldrich, Co (St. Louis, MO, USA).

### Codon optimization, gene synthesis and cloning

A synthetic version of each gene used in this work was designed as previously described [29, 30] where a codon randomization algorithm with the Optimizer software and a codon table containing a fractional preference for each codon equal to that found in the genome of *E. coli* W3110 was used. DNA sequences were designed according to the amino-acidic sequences of the following proteins: BGPh from *Pyrococcus horikoshii* (WP_010884453.1), BGPf from *Pyrococcus furiosus* (WP_011011560.1), BGTl from *Thermococcus litoralis* (WP_004069094.1), BGTk from *Thermococcus kodakarensis* (WP_011250778.1), BGTs from *Thermococcus sibiricus* (WP_015848865.1), BGAb from *Aciduliprofundum boonei* (WP_008082059.1) and BGTb from *Thermococcus barophilus* (WP_013467240.1). DNA sequences were synthesized by Genscript (USA), cloned into pET28a at NdeI and EcoRI restriction sites, and verified by sequencing. In all the cases*, E. coli* DH5α was used for cloning. Codon optimized genes were deposited in GenBank under the accession numbers listed in Table 1.

### Culture growth, gene expression and purification

For the expression of heterologous proteins, *E. coli* BL21(DE3) strains harboring the corresponding plasmids were grown at 37 °C in shake flasks in LB medium in the presence of 50 mg/L kanamycin for plasmid maintenance. Overnight cultures were diluted 1:100 in fresh medium and grown to an OD_600_ of 0.5 before the addition of IPTG to a final concentration of 0.5 mM. Induction was allowed to proceed for 5 h at 22 °C. The cells were harvested, resuspended in 50 mM phosphate buffer pH 6.8, 150 mM NaCl, and 1 % Triton X-100, and disrupted by sonication. Soluble and insoluble fractions of cell lysates were analyzed by SDS–PAGE [31]. After disruption, crude extracts were clarified by centrifugation during 30 min at 15,000*g*. Enzymes were purified by affinity chromatography, using Ni^2+^-NTA Agarose resin (Invitrogen) according to the protocol supplied by the manufacturer. Elution fractions containing the corresponding recombinant enzyme were diafiltrated using an ultrafiltration hollow fiber cartridge (UFP-10-E-3-MA, GE Healthcare Bio-Sciences AB, Sweden) to obtain final protein concentration of 1 mg/mL in ammonium acetate buffer pH 5.5. Protein concentration was determined by the method of Bradford [32], using BSA as standard. Coomassie Brilliant Blue staining was utilized to reveal SDS–PAGE.

### Enzymatic hydrolysis of SG in buffer

A 5 mg/mL SGs stock solution was prepared in 3:1 THF:H_2_O. 60 µl of this solution (final SG concentration: 100 ppm) was added to 3.0 mL of 50 mM phosphate buffer pH 6.5 for BGTl or 3.0 mL of 50 mM citrate buffer pH 5.5 for LacS in a 5 mL capped vial, in the presence of 0.1 % Triton X-100 to help SGs dissolution. The reaction was started by the addition of 14 µg of enzyme/mL buffer and incubated at 70 °C.

### Enzymatic hydrolysis of SG in distilled and commercial crude Biodiesel

13 g of commercial crude biodiesel with a known SGs concentration, or distilled biodiesel spiked with 100 ppm of SGs were added with 1.95 mL of buffer with different pHs and concentrations containing NaCl and BGTl or LacS enzyme. The mixture was thoroughly agitated at 400 rpm in a VP 710 magnetic tumble stirrer (V&P Scientific, San Diego, USA) at different temperatures to optimize the reaction conditions. At different incubation times, 500 μL aliquots were taken for glucose quantification.

The best reaction conditions determined in these assays were used to remove SG from biodiesel at pilot (15 L) and industrial (20 ton) scale, as detailed in the Results section.

### Analysis of SGs hydrolysis

SGs extent of hydrolysis was determined by the formation of glucose, one of the reaction products, as previously described [33]. Since the amount of glucose released from SGs in water/biodiesel emulsions is quantitatively partitioned to the water phase, this method could be used both in aqueous and water/biodiesel reactions. Glucose was specifically converted to gluconate 6-phosphate in a coupled enzymatic reaction (hexokinase, glucose 6-phosphate dehydrogenase), with formation of NADPH, which was determined fluorometrically at 340 nm in a Synergy 2 Multi-Mode Microplate Reader (BioTek). A calibration curve was obtained by measuring known amounts of glucose.

SGs hydrolysis was also analyzed by a SPE-GC-FID based determination previously described [33]. Biodiesel samples were spiked with 50 ppm of cholesteryl glucoside (Sigma 28609) as internal standard.

### β-Glucosidase activity assay

β-Glucosidase activity was determined using a modification of the assay described by Hang et al. [34]. The reaction mixture (500 μL) contained 465 μL of sodium phosphate buffer (25 mM, pH 6.5), 25 μL of 20 mM pNPG, and 10 μL of the appropriate dilution of enzyme-containing sample. After incubation at 80 °C for 5 min, the reaction was stopped by adding 1 mL of cold 200 mM sodium carbonate. The activity of the enzymes was estimated spectrophotometrically by reading the absorbance of the liberated *p*-nitrophenol at 405 nm (*ε* = 18,700 M^−1^ cm^−1^). One unit (U) was defined as the amount of enzyme required for the hydrolysis of 1 μmol pNPG/min, under the assay conditions.

### Biodiesel quality tests

Total Contamination (TC), Cold Soak Filtration Test (CSFT), Filter Blocking Tendency (FBT), and Cold Soak Filter Blocking Tendency (CSFBT) tests were performed according to the procedures described in the EN 12662, ASTM D7501, ASTM D2068 and CAN/CGSB-3.524-2011 norms, respectively, in a laboratory accredited based on the ISO 17025 standard. All experiments were carried out in triplicates.

## Additional files

10.1186/s13068-015-0405-x Molecular modeling of BGTl based on the X-ray structure of BGPh (PDB 1VFF). Left, accessible surface representation of the protein showing the hydrophobic patch colored in gray. Right, electrostatic potential surface calculated using APBS. The positive isosurface is colored blue and the negative surface is shown in red, both calculated at the same potential. A positive electrostatic potential surrounds the hydrophobic parch. The model is shown in cartoon representation.
10.1186/s13068-015-0405-x Effect of temperature on BGTl stability in commercial soybean oil biodiesel/water mixtures. β-Glycosidase activity recovered from aqueous phase during the time course of BGTl catalyzed hydrolysis of SG. Error bars show the standard deviation of three independent assays.
10.1186/s13068-015-0405-x Effect of agitation on BGTl stability in commercial soybean oil biodiesel/water mixtures. β-Glycosidase activity recovered from aqueous phase during the time course of BGTl catalyzed hydrolysis of SG in a 15 L reactor at different agitation speeds. Error bars show the standard deviation of three independent assays.

## Abbreviations

BGAb: β-glycosidase from *Aciduliprofundum boonei*
BGPf: β-glycosidase from *Pyrococcus furiosus*
BGPh: β-glycosidase from *Pyrococcus horikoshii*
BGTk: β-glycosidase from *Thermococcus kodakarensis*
BGTl: β-glycosidase from *Thermococcus litoralis*
BGTb: β-glycosidase from *Thermococcus kodakarensis*
BGTs: β-glycosidase from *Thermococcus sibiricus*
CSFBT: cold soak filter blocking tendency
CSFT: cold soak filtration test
FBT: filter blocking tendency
G6PDH: glucose-6-phosphate dehydrogenase
GC: gas chromatography
HK: hexokinase
IPTG: isopropyl-β-d-thiogalactopyranoside
FID: flame ionization detector
NTA: nitrilotriacetic acid
PCR: polymerase chain reaction
PGPR: polyglycerol polyricinoleate
pNPG: *p*-nitrophenyl β-d-glucopyranoside
SDS-PAGE: sodium dodecyl sulfate polyacrylamide gel electrophoresis
SGase: steryl glycosidase
SGs: steryl glucosides
SMGs: saturated monoacyl glycerides
SPE: solid-phase extraction
TC: total contamination
