# Supplementary figures and images for: An industrial scale process for the enzymatic removal of steryl glucosides from biodiesel
Source: Biotechnol Biofuels. 2015 Dec 21;8:223. doi: 10.1186/s13068-015-0405-x (PMC4687101; doi:10.1186/s13068-015-0405-x)

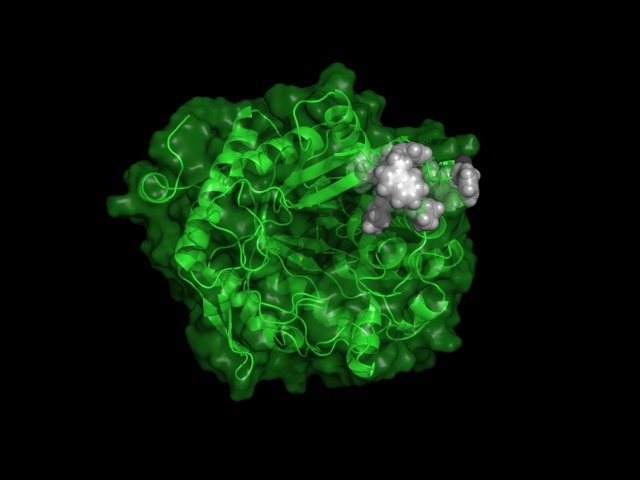

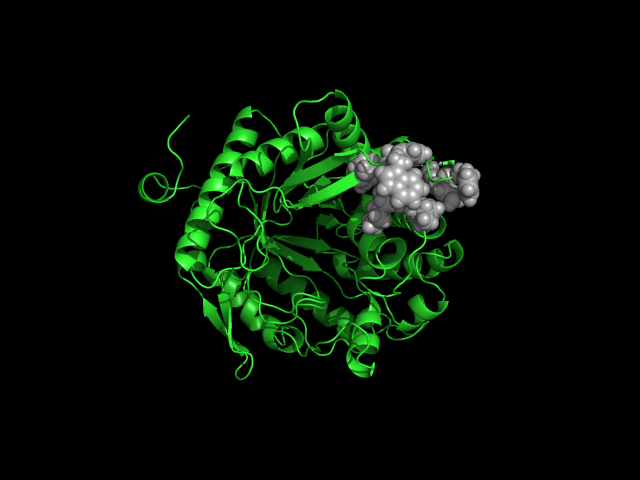

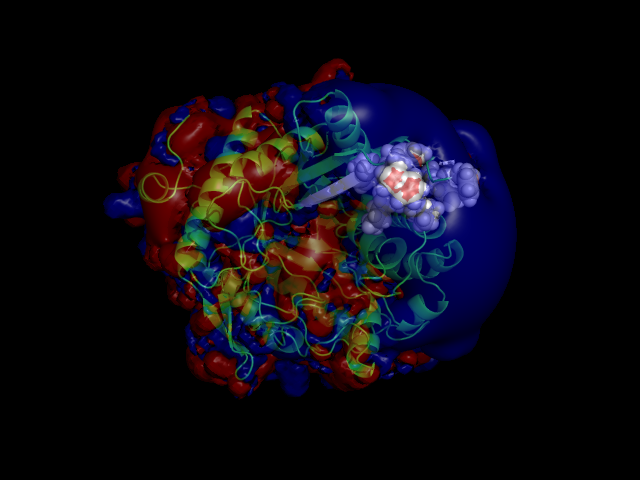

Supplement: Supplementary file 1 — 10.1186/s13068-015-0405-x Molecular modeling of BGTl based on the X-ray structure of BGPh (PDB 1VFF). Left, accessible surface representation of the protein showing the hydrophobic patch colored in gray. Right, electrostatic potential surface calculated using APBS. The positive isosurface is colored blue and the negative surface is shown in red, both calculated at the same potential. A positive electrostatic potential surrounds the hydrophobic parch. The model is shown in cartoon representation. [file 13068_2015_405_MOESM1_ESM.docx]
