# Supplementary material for: An industrial scale process for the enzymatic removal of steryl glucosides from biodiesel
Source: Biotechnol Biofuels. 2015 Dec 21;8:223. doi: 10.1186/s13068-015-0405-x (PMC4687101; doi:10.1186/s13068-015-0405-x)
Supplement: Supplementary file 2 [file 13068_2015_405_MOESM2_ESM.docx]

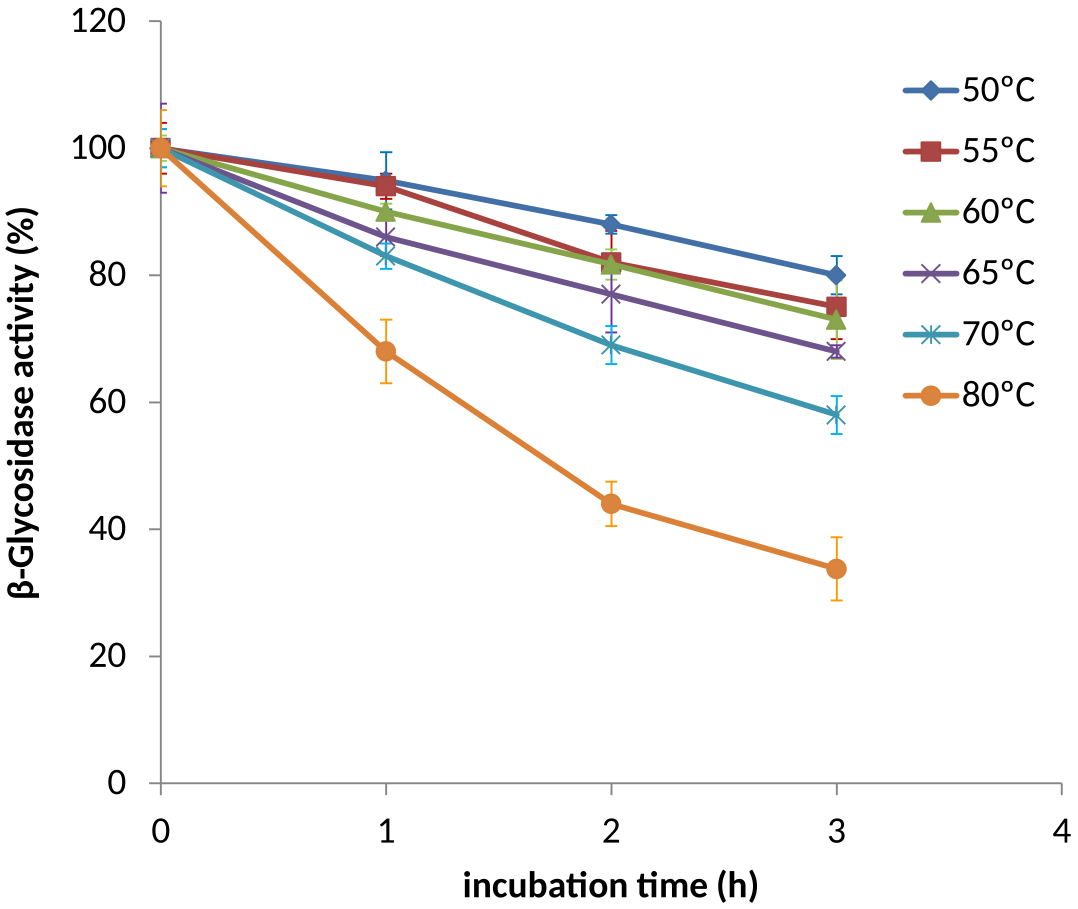

β-Glycosidase activity (%)
incubation time (h)
0
20
40
60
80
100
120
0
1
2
3
4
50°C
55°C
60°C
65°C
70°C
80°C
