# Supplementary material for: An industrial scale process for the enzymatic removal of steryl glucosides from biodiesel
Source: Biotechnol Biofuels. 2015 Dec 21;8:223. doi: 10.1186/s13068-015-0405-x (PMC4687101; doi:10.1186/s13068-015-0405-x)
Supplement: Supplementary file 3 [file 13068_2015_405_MOESM3_ESM.docx]

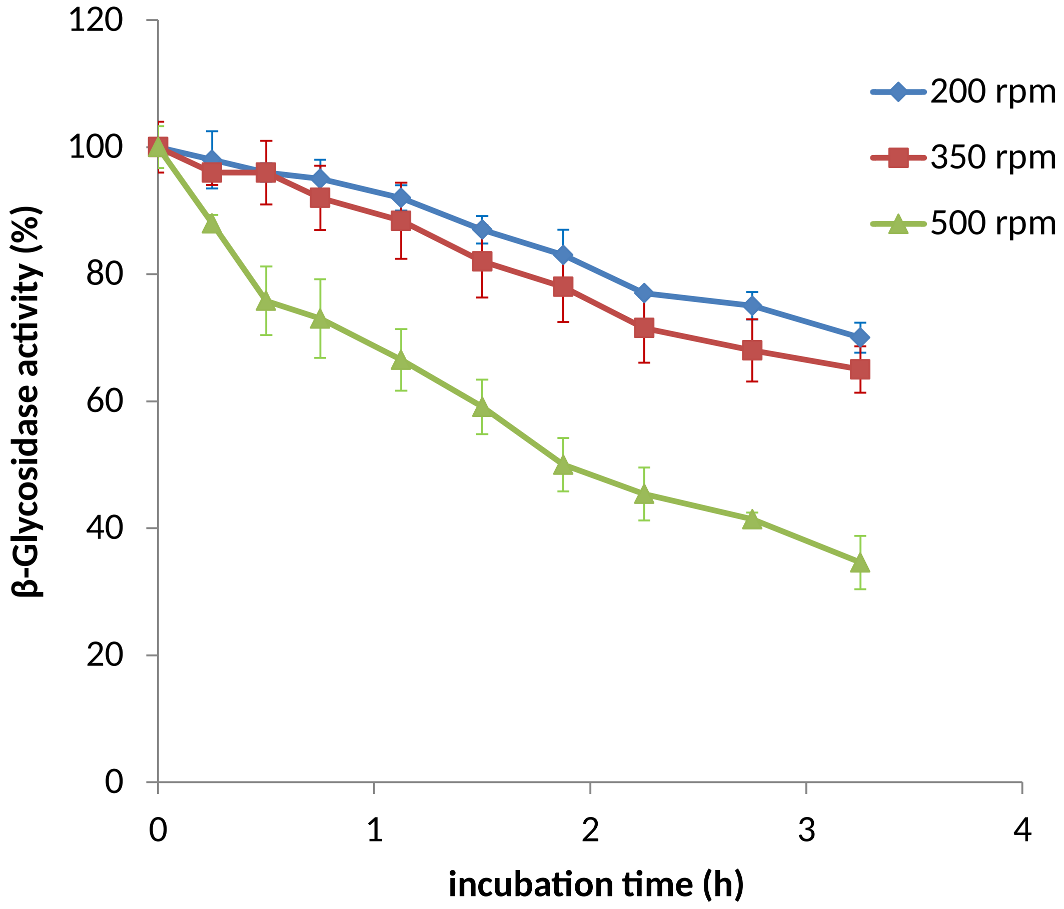

120
100
80
60
40
20
0
0
1
2
3
4
β-Glycosidase activity (%)
incubation time (h)
200 rpm
350 rpm
500 rpm
